# P-819. You get a nudge, and you get a nudge! The impact of a microbiology comment on the treatment of Streptococcus pneumoniae bloodstream infections

**DOI:** 10.1093/ofid/ofaf695.1027

**Published:** 2026-01-11

**Authors:** Nathan Everson, Rachel M Kenney, Geehan Suleyman, Anita Shallal, Robert Tibbetts, Michael Akon, Sydney Vandorf, Michael P Veve

**Affiliations:** Henry Ford Hospital, Detroit, MI; Henry Ford Hospital, Detroit, MI; Henry Ford Health, Detroit, Michigan; Henry Ford Hospital, Detroit, MI; Henry Ford Health, Detroit, Michigan; Henry Ford, Detroit, Michigan; Henry Ford Health, Detroit, Michigan; Eugene Applebaum College of Pharmacy and Health Sciences, Detroit, MI

## Abstract

**Background:**

Bloodstream infection (BSI) occurs in 25-30% of pneumococcal disease. For nonmeningeal *S. pneumoniae* infections, ampicillin or penicillin demonstrate 97% susceptibility on local antibiograms but are not commonly prescribed. The study purpose was to assess the impact of a microbiology nudge comment on narrow-spectrum antibiotic use in patients with *S. pneumoniae* BSI.

**Figure:**
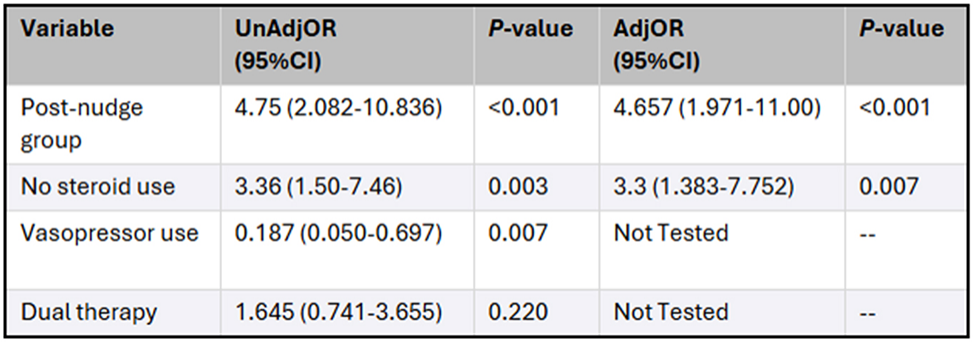
Variables associated with de-escalation to narrow-spectrum at 48-hrs in patients with Streptococcus pneumoniae BSI.

**Methods:**

This was an IRB-approved, single pre-, post-test, quasi-experiment conducted at a five-hospital health system from 10/1/2022 to 9/26/2023 (pre-nudge) and 0/1/2023 to 9/26/2024 (post-nudge). On 9/27/2023, a microbiology nudge was appended to rapid blood polymerase chain reaction (PCR) results: "Drugs of choice = Ampicillin IV or Penicillin IV. For meningitis, use max dose Ceftriaxone IV plus Vancomycin IV until susceptibilities known". Inclusion: hospitalized adults >18 years with confirmed *S. pneumoniae* BSI. Exclusion: IgE-mediated or severe cutaneous β-lactam allergy, polymicrobial infection, comfort/hospice care, death <48-hrs of culture, suspected/confirmed meningitis, or endocarditis. The primary outcome was de-escalation to narrow-spectrum antibiotics within 48-hrs of rapid blood PCR results, defined as IV penicillin or IV ampicillin. Secondary outcomes included all-cause mortality, in-hospital mortality, duration of broad-spectrum antibiotics, and length of hospital/ICU stay.

**Results:**

105 patients were included; 54% pre-nudge, 51% post-nudge. Narrow-spectrum antibiotic use at 48-hrs increased from 29.6% to 66.7% (*P*< 0.001), oral switch increased from 35.2% to 56.9% (*P*=0.026). Broad spectrum antibiotic use decreased from a median (IQR) of 8 (4-12) to 3 days (1-9) (*P*< 0.001). Secondary outcomes including hospital length of stay, 30-day all-cause mortality, and 30-day all-cause readmission, were not different between groups. Median (IQR): 8 (5-16) vs. 6 (4-10) days (*P*=0.083); 1.9% vs 7.8% (*P*=0.150); and 16.7% vs. 7.8% (*P*=0.170) respectively. After adjustment for steroids, the post-nudge group was independently associated with narrow-spectrum de-escalation (Table 1).

**Conclusion:**

Implementation of a microbiology nudge was associated with an increase in 48-hr de-escalation to narrow-spectrum for *S. pneumoniae* BSI, without an observed increase in negative clinical outcomes.

**Disclosures:**

All Authors: No reported disclosures

